# A comprehensive evaluation of the sl1p pipeline for 16S rRNA gene sequencing analysis

**DOI:** 10.1186/s40168-017-0314-2

**Published:** 2017-08-14

**Authors:** Fiona J. Whelan, Michael G. Surette

**Affiliations:** 1 Department of Biochemistry and Biomedical Sciences, McMaster University, 1280 Main St. W, Hamilton, Canada; 2 Department of Medicine, McMaster University, 1280 Main St. W, Hamilton, Canada

**Keywords:** 16S rRNA gene sequencing, Marker gene sequencing, Microbiome, Pipeline

## Abstract

**Background:**

Advances in next-generation sequencing technologies have allowed for detailed, molecular-based studies of microbial communities such as the human gut, soil, and ocean waters. Sequencing of the 16S rRNA gene, specific to prokaryotes, using universal PCR primers has become a common approach to studying the composition of these microbiota. However, the bioinformatic processing of the resulting millions of DNA sequences can be challenging, and a standardized protocol would aid in reproducible analyses.

**Methods:**

The short-read library 16S rRNA gene sequencing pipeline (sl1p, pronounced “slip”) was designed with the purpose of mitigating this lack of reproducibility by combining pre-existing tools into a computational pipeline. This pipeline automates the processing of raw 16S rRNA gene sequencing data to create human-readable tables, graphs, and figures to make the collected data more readily accessible.

**Results:**

Data generated from mock communities were compared using eight OTU clustering algorithms, two taxon assignment approaches, and three 16S rRNA gene reference databases. While all of these algorithms and options are available to sl1p users, through testing with human-associated mock communities, AbundantOTU+, the RDP Classifier, and the Greengenes 2011 reference database were chosen as sl1p’s defaults based on their ability to best represent the known input communities.

**Conclusions:**

sl1p promotes reproducible research by providing a comprehensive log file, and reduces the computational knowledge needed by the user to process next-generation sequencing data. sl1p is freely available at https://bitbucket.org/fwhelan/sl1p.

**Electronic supplementary material:**

The online version of this article (doi:10.1186/s40168-017-0314-2) contains supplementary material, which is available to authorized users.

## Background

The recent surge of next-generation sequencing technologies have allowed the scientific community to use marker genes, most popular of which being the 16S rRNA gene, to more thoroughly understand mixed bacterial communities (i.e., microbiomes). However, the adoption of any new technology requires standards and quality control. Alongside a plethora of 16S rRNA gene amplicon studies, quality control efforts have addressed the standardization of experimental and bioinformatic methods. For example, laboratory standards have been proposed for the preparation and storage of biological samples [1–3] as well as procedures for the isolation and sequencing of DNA which mitigate environmental contamination [4, 5]. Sequencing controls have greatly reduced variability between laboratories and datasets [5]. Similarly, efforts have been made to standardize the bioinformatic processing of amplicon sequencing results [6, 7]. Next-generation sequencing technologies are subject to varying levels of sequencing error; traditionally, processing of amplicon sequencing data has involved filtering based on input sequence quality, followed by clustering of sequences into operational taxonomic units (OTUs) which are given a taxonomic label based on their similarity to a known database (for e.g. [8–10]). Choice of algorithms for quality filtering, OTU clustering, and taxonomic assignment have been shown to affect the downstream analysis of biologically meaningful results [11].

OTU clustering, typically computed at 97% sequence similarity, can be divided by approach. Reference-based (or phylotyping) approaches, such as BLAST [12] and UCLUST-reference [13], compare input sequences to a reference database. In contrast, de novo-based approaches are independent of a reference set. De novo approaches include hierarchical clustering methods such as Mothur’s average linkage algorithm [7], and ESPIRIT [14], as well as greedy algorithms such as CD-HIT [15, 16], DNACLUST [17], UPARSE [18], and AbundantOTU+ [19]. Similarly, choice of taxonomic assignment algorithm and reference database also vary across 16S rRNA amplicon studies.

Recent benchmark studies have helped identify some of the most accurate methods in each of these categories. For example, Kopylova et al. identified a series of clustering methods, including UPARSE and USEARCH, which outperformed the widely used UCLUST algorithm [11]. Schloss and colleagues have also presented numerous comparisons of OTU clustering algorithms to find that de novo methods out perform reference-based methods [20, 21] and, more specifically, that the average neighbour algorithm often outperforms all others [20, 22, 23]. Some comparisons of taxonomic methods have also been performed (for e.g., [24]).

Without a comprehensive workflow, such a surplus of available methods for 16S rRNA gene data processing makes it difficult to identify the most accurate approaches. Further, because each step has been developed independently, processing often involves file and command line manipulations between steps; conducting these manipulations in high-throughput is often inaccessible to a traditionally trained microbiologist, and makes it difficult to reproduce or extend data analyses. Widely used and important tools, such as QIIME [6] and Mothur [7], have aided in these issues; however, their step-by-step approach and various parameters represent a significant barrier to effective amplicon data processing and do not fully mitigate issues of reproducibility. To combat this need for ease-of-use, reproducible data processing, and want of a non-biased assessment of processing options, we developed the short-read library 16S rRNA gene sequencing pipeline (sl1p, pronounced “slip”), a 16S rRNA data processing software. sl1p takes Illumina-generated FASTQ files as input and automates all data processing to generate a reproducible OTU table with taxonomic assignments. This pipeline is compatible with any primer set or amplicon gene, and currently offers access to eight OTU clustering algorithms, two taxonomic assignment options, three 16S rRNA gene reference databases, and two phylogenetic outputs. As presented here, the default processing steps and software used in sl1p have been determined to be the most accurate available approaches based on their assessment with synthetic communities generated as part of the Human Microbiome Project (HMP) [25], and a set of 190 individually picked isolates. All steps in data processing are recorded by sl1p in a log file for future reference and reproducibility.

sl1p is a tool designed to be accessible to the microbiologist without detailed bioinformatic training; as such, it is fully automated, needing one line input from the user upon startup. Further, the output of sl1p includes an R markdown file with the appropriate code to visualize read counts per sample, taxonomic assignments, *α*-, and *β*-diversity from which the user can begin their own analyses. sl1p is freely available at https://bitbucket.org/fwhelan/sl1p.

## Methods

### The sl1p pipeline

**Fig. 1:**
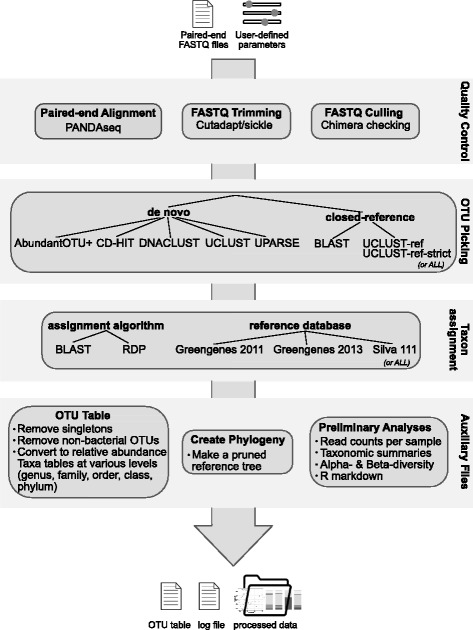
Schematic of the sl1p pipeline. The user input consists of FASTQ files and processing parameters. Upon input, the user can choose to deviate from the default parameters to choose from various options for OTU picking algorithms, taxonomic assignment, and reference database. Every step that sl1p utilizes is recorded in log and error files for the purposes of debugging, reference, and reproducibility. For more detail, see Additional file 1

sl1p is a data processing pipeline developed for the automated, reproducible, and accurate processing of paired-end amplicon FASTQ data (Fig. 1 and Additional file 1). Input to sl1p includes (a) FASTQ reads in Illumina’s standard FASTQ format, and (b) a ‘file of filenames’ file listing all FASTQ files and their file path. Optionally, the user can also include a sequencing information file if they wish to use primer sets outside of the built in defaults (v3, [26]; v34, [27]; v4 [27–30]). Each step in sl1p’s data processing approach is recorded in a log file, for future reproducibility; further, the standard error output of each step is recorded to an error file to aid in any necessary de-bugging.

During initialization, the user can use command line flags to deviate from sl1p’s default functionality (Additional file 1). By default, quality filtering consists of cutadapt [31] to trim the PCR primers from the FASTQ input, PANDAseq (version 2.9) [32] to align paired-end reads, sickle (https://github.com/najoshi/sickle; version 1.33) to quality trim the resulting pairs, and USEARCH [13], as implemented in QIIME (v1.9.1) [6], to identify and remove chimeric sequences. Users have the choice of eight OTU clustering approaches: five greedy algorithms including AbundantOTU+ 0.93b (default; [19]), CD-HIT 3.1.1 [15, 16], DNACLUST (release 3) [17], UCLUST v1.2.22q [13], and UPARSE (USEARCH version 8.0.1623) [18], and two reference-based approaches, BLAST 2.2.22 [12] and UCLUST [13], which can either be strictly closed (UCLUST-ref-strict) or conduct closed clustering followed by de novo on any leftover sequence not matching the reference database (UCLUST-ref). Taxonomic assignment (and OTU clustering, where appropriate) can be assigned using two methods, BLAST or the RDP Classifier 2.2 (default; [33]), against three reference databases: Greengenes Feb. 2011 (default), Greengenes Aug. 2013 [34], and Silva Release 111 [35]. Finally, OTU tables, phylogenies, and preliminary analyses are conducted using QIIME and R (v3.3.1). Importantly, as part of sl1p’s commandline options, the user can choose to run all possible combinations of OTU clustering algorithms, taxonomic assignment methods, and choice of reference databases automatically with one command, making comparisons of available methods reproducible and easy to approach.

The sl1p pipeline is open source and publicly available at https://bitbucket.org/fwhelan/sl1p. The pipeline is written in Perl and consists of one main script which calls on auxiliary scripts to aid in reformatting data between steps as necessary. Accompanying setup and install scripts are provided to download and install sl1p.

### Generation of test datasets

#### The Human Microbiome Project Mock Communities (HMP-mock)

Genomic DNA of two unique representations of a 20 member mock community generated as part of the Human Microbiome Project [25] were obtained from BEI Resources (Catalog Nos. HM-782D and HM-783D; ATCC, Manassas, VA). The first sample (HMP-mock1) is an even distribution of the 20 bacterial organisms from 17 genera, whereas the second (HMP-mock2) is a staggered distribution of the same organisms [25]. For each sample, 3 PCR replicates were generated by using 1 *μ*l of genomic DNA PCR amplified using 1 *μ*l of dNTPs, 0.25 *μ*l of *Taq* polymerase (Life Technologies, Carlsbad, CA) and 5 *μ*l of PCR primers designed for the v3 region of 16S rRNA gene [26]. These amplification products were then split across two runs of the Illumina sequencer to generate sequencing replicates. Base calling was performed using CASAVA (v1.8.2). Sequencing depth ranged from 5917 to 113,084 reads with an average of 57,257. A negative PCR control was generated in parallel.

#### Single and Combined Isolate Controls (URTCul)

One hundred ninety single colonies were picked from a collection of upper respiratory tract culture isolates (URTCul) and restreaked until pure on appropriate solid agar plates as described in [36]. Once pure, isolates were picked directly into 5% Chelex, boiled, and centrifuged at 13,000 rpm for 5 min. 5 *μ*l of the supernatant was used as template for a 50 *μ*l PCR reaction of the variable regions 8F-926R [37, 38] of the 16S rRNA gene and sequenced using Sanger sequencing (amplicon length = 918 bps). The resulting Sanger sequences for each isolate were taxonomically assigned using independent blastn searches against NCBI’s RefSeq database. Taxonomic assignments were made to the species level; in the case of multiple species matching with percent identity within <1% of each other, multiple species names were included in the taxonomic assignment are presented (e.g. g__Streptococcus;s__infantis_mitis). This dataset contained 8 unique genera and 33 unique species.

**Fig. 2:**
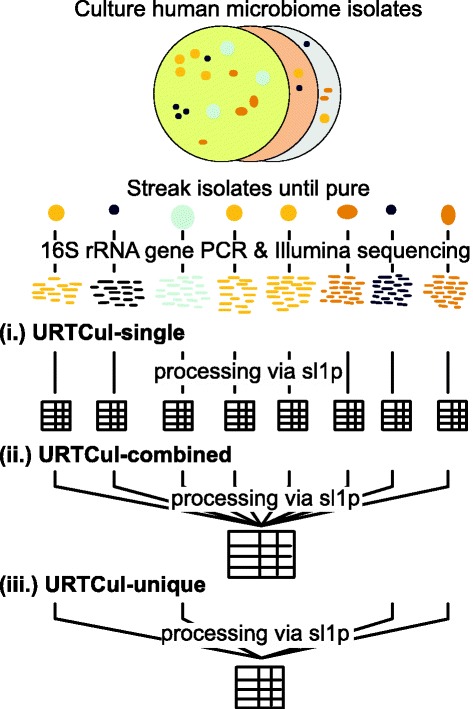
Schematic of URTCul mock community generation. Isolates were individually picked from solid agar plates and amplified using Sanger and Illumina sequencing approaches. Following Illumina sequencing, the resulting reads from each individually sequenced isolate were analyzed individually (**URTCul-singles**), in combination (**URTCul-combined**), or as a combination of each uniquely identified taxa (**URTCul-uniques**)

For Illumina sequencing, PCR amplification of the v34 region (341F-806R, [27]) was performed and sequencing was conducted on an Illumina MiSeq sequencer to produce paired-end, 250 bp reads; each isolate was PCR amplified with its own unique barcoded primer (Fig. 2, URTCul-singles). Because each isolate was uniquely barcoded, resulting reads per sample were expected to have originated from an individual colony. Occassionally, isolates were contaminated with a second, co-occurring organism, resulting in reads from > 1 organism. In these cases, specifically when a sample contained ≥ 15% of reads from 2 taxonomically divergent organisms, the sample was culled; this process resulted in the culling of 9 samples. The average number of sequenced reads per isolates was 12 (range 1–81); because each sample contained only one organism, each sample was designated to 0.01% of an Illumina MiSeq sequencing run.

After amplification and Illumina sequencing of each isolate individually, the raw FASTQ reads were combined in silico to create one sample (Fig. 2, URTCul-combined). Further, the taxonomic assignments of the Sanger sequencing results were consulted to create a second in silico sample in which only uniquely identified taxa were combined (Fig. 2, URTCul-uniques). The artificial sequencing depths of these 2 samples were 2148 and 423, respectively. These data are publicly available (BioProject ID PRJNA 381557).

#### Publicly available dataset

Additionally, a publicly available dataset of human fecal microbiota samples (Bioproject Submission SUB2392090; [39]) was used in testing the phylogenetic outputs of sl1p displayed in Fig. 6.

### Data processing comparisons

**Fig. 3:**
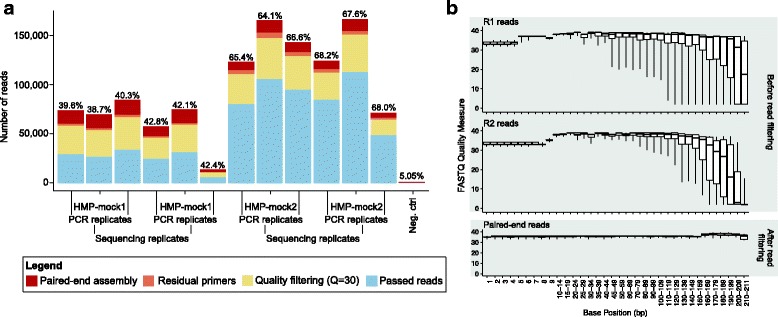
sl1p effectively removes low quality reads. **a** sl1p’s quality control workflow consists of paired-end assembly, removal of residual primers, quality trimming, and length filtering. Here, the number of reads culled at each step is presented. Inline percentages indicate the percentage of raw input reads which remain following the quality control process. **b** This process successfully removes bases of low quality from the resulting paired-end reads as demonstrated here on the raw sequence input from 2 unique mock HMP samples sequenced using 3 PCR and 2 sequencing replicates

All output data processing comparisons were based on OTU tables, map files, and phylogenies generated by sl1p v4.1 using the -p all -d all and -t all flags. All analyses were computed in R using phyloseq [40], ggplot2 [41], and reshape2 [42] with the following exceptions. FastQC [43] was used to calculate FASTQ quality scores used in Fig. 3. Graphlan [44] was used to visualize phylogenies as presented in Fig. 6. All data processing was computed on a standard personal desktop computer running Ubuntu 14.04. The R and Perl code necessary to reproduce these data comparisons are available as an R markdown (Additional file 2) and accompanying HTML output (Additional file 3).

## Results

The short-read library 16S rRNA gene sequencing pipeline (sl1p) was developed as an automated and reproducible 16S rRNA gene sequencing processing tool. The output of this tool consists of an R markdown file and accompanying HTML output showing preliminary analyses of the data (e.g. Additional file 4). In order to determine the most accurate default settings of this tool, we systematically tested various methods within the sl1p workflow using 2 approaches (i.) 2 mock community samples from the HMP (HMP-mock), and (ii.) 190 single bacterial isolates (URTCul-singles) and their combination as a totality of the 190 sequencing results (URTCul-combined) or the combination of unique taxa from this pool (URTCul-uniques).

### sl1p removes low quality reads effectively

One of the consequences of using next-generation sequencing technologies in high-throughput is the propensity for sequencing error. For instance, Illumina technology is known to have an increased error rate towards the 3’ end of the read, and that the reverse read is generally of poorer quality then the forward. Mitigating this error prior to OTU generation and taxonomic assignment is essential in order to refrain from the generation of spurious OTUs.

sl1p utilizes a multi-step approach to quality control. Immediately following removal of sequencing primers with cutadapt, forward and reverse reads are assembled using PANDAseq. While many options are available for the merging of paired-end reads, PANDAseq includes both quality filtering and read assembly. Across our PCR and sequencing replicates of HMP-mock, approximately 12.5% of raw input reads were culled at this step (Fig. 3a); the majority of culled reads were due to mis-alignment of forward and reverse reads. Following, cutadapt was used to remove any reads containing Illumina annealing or sequencing primers. While this step removed only 2.7% of the HMP-mock input (Fig. 3a), we have found it to be an important way of removing erroneous sequencing results, and a measure of an infrequent poor Illumina sequencing run. Next, sickle was used to trim quality sequence (and to remove any reads < 100 bp post-trimming). It is at this stage where the most quality-filtering is done, with an average 29.6% read loss (Fig. 3a). However, it is this strict quality filtering that results in clean, high-quality paired-end reads (Fig. 3b); when we compare this strict threshold with lower quality cutoffs, we begin to see a decline in the final paired-end read quality as the cutoff drops below 30 (Additional file 5).

The last step in sl1p’s quality control workflow is chimera checking. Because 16S rRNA gene amplicon data is generated via PCR amplification, chimeric sequences can be an issue, especially if the PCR amplification reaction traverses a highly conserved region as is the case for multi-variable region amplicons. As such, sl1p uses QIIME’s implementation of USEARCH to conduct chimera checking on the generated paired-end reads (Fig. 1 & Additional file 1). This approach is database-dependent; however no significant differences were observed between sl1p’s 3 options for reference database (removal of 0.36%, 0.4%, and 0.39% of reads for Greengenes 2011, 2013, and Silva Release 111, respectively on the HMP-mock data).

Following sl1p’s quality control workflow, an average of 55.2% of the raw input HMP-mock reads remain. This percentage is higher to that found with the URTCul dataset (mean of 30.4%); a greater number of unassembled paired-end reads (57.9% of raw input removed during PANDAseq alignment) were observed with the URTCul v34 sequencing, possibly due to the shorter overlap in the target sequence (Additional file 6).

### OTU clustering algorithms produce varying numbers of OTUs compared to known input communities

Clustering of input reads into Operational Taxonomic Units (OTUs) has been the most well-studied effect on processed reads [11, 22, 45–48]. OTUs are typically clustered based on a 97% threshold based upon imperial studies identifying this as the differentiating threshold of species [49]; however when sequencing is restricted to small regions within the gene, this threshold may provide differentiation between the genus and species level, depending on the organism in question [24].

**Fig. 4:**
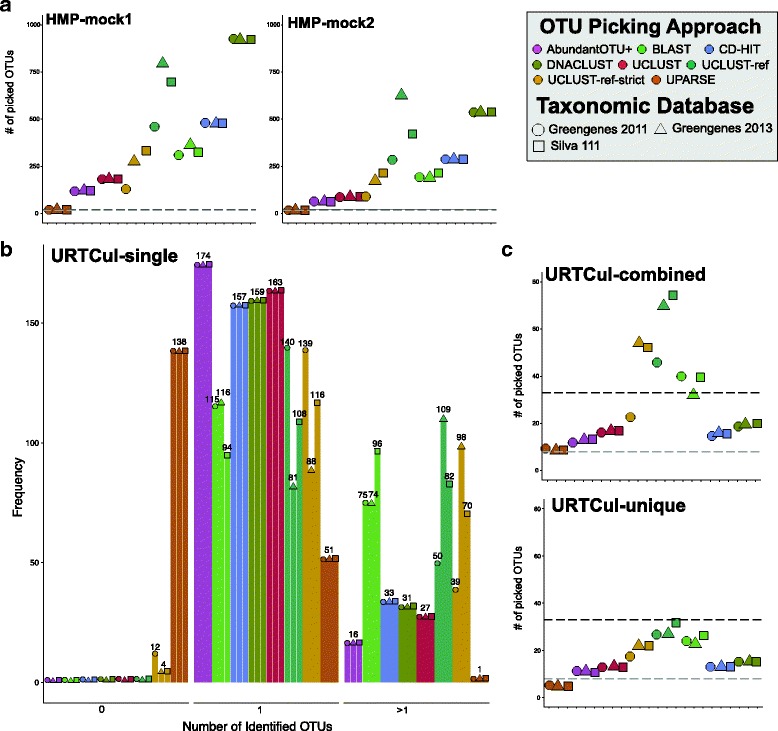
OTU clustering methods perform variably. **a** Eight methods were used on control communities of known composition to report OTU counts compared to known sample diversity (*black dotted lines* = number of genus; *grey dotted line* = number of species). Non-bacterial sequences were removed as part of sequence processing. Similar results were obtained when singletons were also removed (Additional file 5). **b** A group of 190 single isolates were independently sequenced in order to test varying OTU clustering algorithm’s ability to correctly identify 1 OTU within the input sample. **c** When these individual isolates were combined, the number of OTUs generated often lies between the known number of unique genera and species within the samples

sl1p provides 8 OTU clustering approaches from which the user can choose from upon initialization of the pipeline. As expected, *de novo* clustering methods produce observed OTU numbers independent of the reference database, whereas some variability in observed OTUs is seen with reference-based approaches (Fig. 4). Most of these options over-estimate the number of OTUs within the HMP-mock and URTCul datasets when compared to the known taxonomic composition (Fig. 4). This is perhaps the most evident in the HMP-mock dataset where some algorithms, such as DNACLUST, over-estimated sample diversity by almost 40x (Fig. 4a, Additional files 7 and 8). When Swarm [50], USEARCH v6.1.5.44, and Mothur’s average neighbour (v1.25.0) algorithm were compared using sl1p-generated quality filtered reads, sample diversity was also over-estimated, though the removal of singletons greatly reduced the number of spurious OTUs (Additional file 9). When OTUs with a successively small number of defined reads where culled, the number of observed OTUs quickly converged to the expected community diversity (Additional file 10), suggesting that these spurious OTUs are often due to low abundance reads. Other algorithms, such as UPARSE, under-estimated OTU abundance (Fig. 4, Additional files 7-8 and 10). Of those tested, the approaches which most closely estimated within sample OTU diversity in the HMP-mock samples were AbundantOTU+, UCLUST open reference picking, and UPARSE.

Within the URTCul-single dataset, in which each sample consisted of DNA from a single bacterial colony, many OTU picking algorithms over-estimated sample diversity in multiple samples (Fig. 4b). UPARSE, with its own approach to sequence quality control (Additional file 1), often underestimated sample diversity. However, many approaches, including AbundantOTU+, CD-HIT, DNACLUST, and UCLUST often identified the sole OTU within the sample (Fig. 4b). When these individually sequenced isolates were combined, most OTU picking approaches estimated sample diversity between the known number of genera and species present within the samples (Fig. 4c). Notably, UPARSE under-estimated diversity, generating 9 and 5 OTUs in the URTCul-combined and -unique samples, which consisted of 33 species from 8 genera. As next-generation sequencing approaches become more accessible to this field, the feasibility of implementing these methods on a common laboratory desktop is increasingly more practical and should be considered (Table 1).

**Table 1:** CPU time for OTU clustering approaches implemented in sl1p

OTU picking approach	CPU time (in mins)
AbundantOTU+	3.38
BLAST	127.17
CD-HIT	13.32
DNACLUST	0.08
UCLUST	0.21
UCLUST-ref	0.69
UCLUST-ref-strict	0.82
UPARSE	0.28

All calculations were computed on a standard Desktop running Ubuntu 14.04

### Choice of data processing algorithms affect taxonomic assignment

**Fig. 5:**
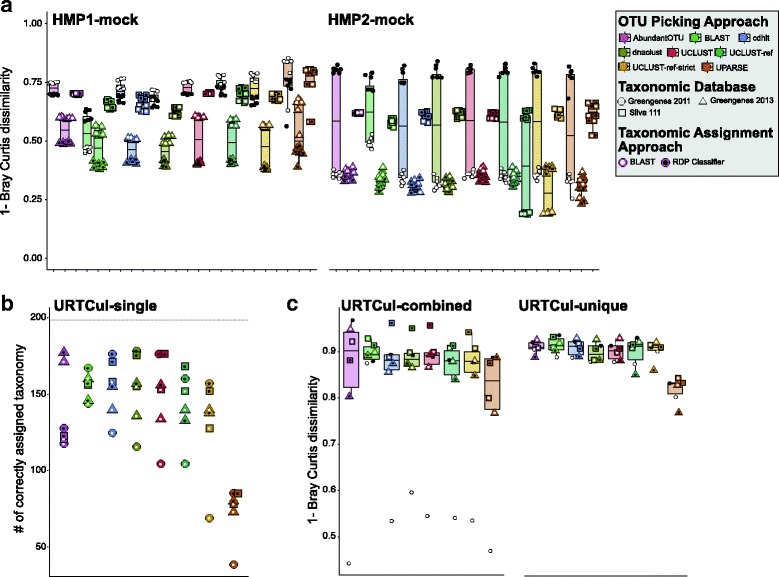
Taxonomic assignment is dependent on up-stream choices in 16S rRNA gene processing. sl1p implements 2 methods of taxon assignment across 3 reference databases. By running all methods, we compared taxon assignment against expected control samples. **a** The negated Bray-Curtis dissimilarity was used to identify which taxonomically assigned OTU sets most closely matched the known composition of the mock HMP communities (**a**) and the combined URTCul isolates (**c**). **b** In a set of 190 single isolate samples, the number of samples whose most abundant OTU correctly matched full-length 16S rRNA Sanger sequencing results is displayed

However, as has been previously addressed [47], what is more important than simply the number of OTUs produced is how the taxonomic assignment and corresponding relative abundance of each taxa compares to the known sample composition. To measure this, we compared the known composition of the mock datasets to the OTU composition generated via sl1p’s options for OTU clustering, taxon assignment, and reference database (Fig. 5). The processing options which showed the most similarity to a given mock community was highly sample-dependent; for example, a combination of UPARSE, BLAST, and reference database Greengenes 2011 showed the most similarity to the HMP-mock1 sample whereas AbundantOTU+ and the RDP Classifier replaced UPARSE and BLAST as the most accurate OTU picking algorithm and taxonomic assignment method in HMP-mock2 and URTCul-combined (Fig. 5a, c). Further, the combination which produced the most similar output to the known composition of HMP-mock1 (UPARSE, BLAST, and Greengenes 2011) produced one of the least similar outputs in URTCul-combined (Fig. 5a, c). In the URTCul-singles dataset, the most abundant OTU’s taxonomic assignment was compared with the results of taxonomic assignment based on full-length Sanger sequencing of the 16S rRNA gene. In this dataset, the RDP Classifier produced the highest number of correctly assigned taxa accompanied with either Greengenes 2011 or the Silva database (Fig. 5b). These results indicate the impact of sample composition as well as choice of OTU picking approach, taxon assignment method, and reference database on the underlying biological implications of these data.

**Fig. 6:**
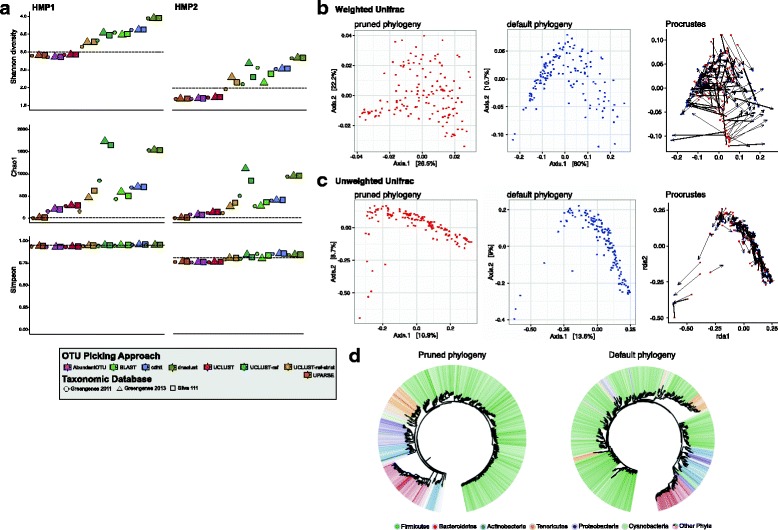
Analyses of biologically-meaningful outputs are dependent on 16S rRNA sequence processing. **a** *α* diversity metrics vary greatly between OTU picking approaches, and are dependent on the choice of reference database in the case of reference-based OTU clustering methods. *Black dotted lines* indicate the expected values of these metrics based on known community composition. **b-c** Phylogeny-dependent *β* diversity metrics, including Weighted UniFrac (**b**) and Unweighted UniFrac (**c**), differ depending on the method of phylogeny-generation. A comparison of the distribution of samples via a Procrustes analysis indicates the impact that the phylogenetic tree makes on these data. **d** sl1p generates 2 phylogenies. The default phylogeny represents the phylogeny generated as part of the default QIIME workflow. The pruned phylogeny is generated by sl1p by pruning the Greengenes reference phylogeny to those branches which are present within the sample set

To further quantify these differences, comparisons can be made between the known taxa and relative abundance compared to each set of OTU picking, taxonomic assignment, and reference database options (Additional files 11 and 12). At this level of resolution, independent of the number of OTUs assigned to each genera, we can see that the proportions of each genera output from sl1p reflect the expected proportions in each of the HMP-mock samples. However, in some sets of processing options, some mistakes are made in taxonomic assignment. The combination of the RDP Classifier and Greengenes 2013 database, for example, incorrectly identifies genus *Flexispira* in place of the *Heliobacter* genus in HMP-mock1 (Additional file 11). In other cases, the correct assignment is made, though more conservatively left at the family, order, or class level (Additional files 11 and 12); for example, Greengenes 2013 using BLAST as the taxon assignment algorithm assigns some OTUs to the class *Bacilli*, failing to differentiate between the *Bacillus, Listeria, Staphylococcus, Enterococcus,* and *Streptococcus* species present in HMP-mock1. Overall, across all methods and the HMP-mock samples, BLAST in combination with Greengenes 2011 was the only combination to provide no errors in taxonomic assignment at the genus level. This accuracy comes with a small increase in computing time compared with the RDP Classifier (data not shown).

### Choice of processing methods affect biologically relevant results of 16S rRNA gene sequencing

Like all bioinformatic pipelines and processing workflows, what is most important in the output is the reflection of the true underlying biology in the results. While 16S rRNA sequencing data can be analyzed in a number of ways in order to answer many unique research questions, calculations of *α* and *β* diversity are often fundamental to analyses. *α* diversity, or within sample diversity, is a calculation performed on each sample within a dataset. This metric can be calculated using different indices depending on the question at hand. Popular approaches include Shannon and Simpson diversity as these indices incorporate both evenness and richness of the community into their calculations [51, 52]. Other metrics, such as Chao1, are estimates of species richness [53]. Using output of the sl1p processing pipeline, we calculated the Shannon, Chao1, and Simpson diversity metrics on the HMP-mock data (Fig. 6a & Additional file 13). Here, only the OTU clustering algorithm contributes to the estimated richness and evenness of OTU composition, except in the case of reference-based algorithms which are database-dependent (Additional file 1). We observe that the output of *α* diversity metrics is dependent on the processing methods employed. The range of calculated Shannon diversity scores within the same sample processed using different commonly-used approaches is greater than 1.0 (range 1.54-2.84) (Fig. 6a). Similarly, Chao1 estimates species richness anywhere from 20 to 2,451 depending on data processing options employed; Simpson diversity, in contrast, has much less observed variability between OTU clustering methods and reference database choice. Interestingly, these metrics are also affected by changes in read depth as seen in the variation between sequencing replicates (Additional file 13a); rarefaction of reads somewhat reduces this variation depending on the metric employed (Additional file 13b).

*β*, or between-sample, diversity is often used as a measure of difference between sample states (e.g. health and disease). Similar to *α* diversity, there are a variety of distance metrics one can utilize depending on the question at hand. A popular set of these metrics use the phylogenetic distances between OTUs as a contributor to the distance score. Using sl1p, we discovered that the output of these metrics are dependent on how the accompanying phylogenetic tree is generated Fig. 6b-c). Comparisons using Procrustes analysis show substantial differences in the PCoA plots generated using the weighted UniFrac method with different phylogenetic inputs (Fig. 6b-c). One approach recommended in the QIIME workflow, is the use of PyNAST [54] and FastTree [55] to create a multiple sequence alignment and phylogeny of the representative sequence from each OTU in the community (Fig. 6d, default phylogeny). However, because this phylogeny is reliant on the sequence diversity within the sequenced variable region, which is often ≤100–300 bp in length, it often does not reflect the true bacterial phylogeny but instead creates paraphyletic phyla (Fig. 6d). Because of this, sl1p generates an alternate phylogeny which represents the Greengenes reference 16S rRNA gene phylogeny trimmed to those OTUs present within the given dataset. Beginning with a curated phylogeny ensures that the phylogenetic relationships between organisms within a given sample set are preserved. Using these phylogenies to generate the Weighted and Unweighted UniFrac metrics, summarized here as Principal Coordinate Analyses (PCoAs), results in differences in the calculated distance between the samples within this community (Fig. 6b-c); Procrustes analysis was used to visualize the differences between these phylogenetic inputs. These results indicate that processing options greatly affect the output and potential interpretation of 16S rRNA gene sequencing results.

## Discussion

sl1p is an automated, reproducible 16S rRNA gene sequencing processing pipeline that makes 16S rRNA data processing accessible to those without formal bioinformatic training. sl1p is not restricted by variable region or choice of PCR primer set. In this study, we outline the workflow of this tool, which can be broken down into 3 main steps: FASTQ quality control, OTU clustering, and taxonomic assignment (Fig. 1). We show how sl1p can aid in the comparison of multiple options and the effects they have on downstream analyses. The quality control workflow within sl1p was determined based on the parameters necessary to obtain high quality base pair assembly along the length of each paired-end sequence (Fig. 3). In order to compare the effect of various OTU picking approaches and taxonomic assignment methods, mock communities were employed. Comparisons of OTU clustering algorithms displayed a wide range of predicted OTUs, generally over-estimating diversity. This, as well as the under-estimations made by UPARSE (Fig. 4), have been previously shown [11, 20, 23]. Further, the choice of taxonomic assignment algorithm and reference database greatly influenced the predicted taxonomic composition of the communities (Fig. 5).

Most importantly, the use of sl1p to compare data processing outputs (OTU tables, taxonomic summaries, and phylogenies) recognizes the effect processing options have on biological analyses (Fig. 6). Popular *α* diversity metrics such as Shannon diversity are greatly affected by OTU clustering options and sequencing depth (Fig. 6a). These results have implications on the interpretation of microbiome studies across manuscripts and research groups which may process their data using different methods. Further, the differences between sequencing runs have implications for studies which are split across multiple sequencing runs due to size of the sample set. Importantly, the rarefaction of these data did not fully mitigate these effects (Additional file 13b). Further, the alternative phylogenetic representation of the OTU data generated by sl1p better describes the bacterial tree of life, allowing for more accurate *β* diversity distances to be calculated between samples, furthering our knowledge of differences between varying microbial communities.

The default parameters of sl1p were carefully chosen based on the analyses presented within this study. Of course, all algorithms and tools tested have their own merits and niches within this widely growing field; this is reflected in the fact that no set of tools out performed others in all circumstances (Figs. 4, 5 and 6). We chose AbundantOTU+ as the default OTU picking approach. AbundantOTU+ most closely predicted the correct number of OTUs within HMP-mock1, HMP-mock2, URTCul-combined, and URTCul-unique, without under-estimating diversity. AbundantOTU+ was also the tool able to correctly predict the highest number of single isolate samples in the URTCul-singles dataset. This method also performed well in tests of correctly identified taxa, including the Bray-Curtis dissimilarity comparisons. For choice of taxon assignment algorithm, we chose the RDP Classifier as sl1p’s default. This tool consistently calculated the most number of accurate URTCul-singles isolates, and out-performed or tied BLAST performance on Bray-Curtis dissimilarity comparisons in all cases except for HMP-mock1. These results mirror previous comparisons of taxonomic assignment as completed by Liu et al. [56]. Lastly, Greengenes 2011 is sl1p’s default reference database based on its superior performance in the Bray-Curtis distance comparisons of the HMP-mock communities and as one of the best choices for genus-level taxon identification for the URTCul data. Even though the Greengenes 2013 reference database represents an update to the 2011 version, it often incorrectly predicted taxonomic assignments to the species level which were incorrect compared to the known composition of the mock communities.

It is important to note that these default parameters are based on mock communities of human-associated microbes and may not represent the best combination of tools in the study of other microbiota. The authors hope that by providing a pipeline in which multiple OTU picking, taxonomic assignment, and reference database options are easily accessible, that the user can choose to easily deviate from these defaults as they see fit. Further, as the field of microbiome research continues to grow new approaches to data processing can be implemented in sl1p and benchmarked against established approaches. Having a non-biased method for tool comparison will be important for the maturation of this field.

## Conclusions

In conclusion, we present a 16S rRNA gene sequence processing workflow with the aim of generating the most biologically meaningful outputs for the furthering of 16S rRNA gene sequencing techniques and microbiome research in general.

## Additional files

Additional file 1 Full schematic of the sl1p software. sl1p is a pipeline script which calls a series of sub-routines based on user-defined parameters given as a series of runtime flags. This Fig. accompanies Fig. 1. (PDF 126 kb)

Additional file 2 R markdown output of the data processing present within this manuscript. This file is in HTML format and displays the output generated by Additional file 3. (HTML 10,148 kb)

Additional file 3 The R markdown file of all data processing present within this manuscript. (RMD 597 kb)

Additional file 4 The R markdown output generated by sl1p for the HMP-mock dataset. (HTML 1546 kb)

Additional file 5 Comparisons of various thresholds for quality trimming. Sickle takes as input a quality threshold with which it determines its quality trimming parameters. Here, we compare the results with a threshold of 30 (Fig. 3) with sequentially lower quality threshold inputs into sickle. (PDF 40 kb)

Additional file 6 Outline of reads lost in the URTCul dataset during sl1p’s quality control pipeline. More input reads were culled during the PANDAseq alignment step in this dataset compared to HMP-mock (Fig. 3), possibly due to a difference in target variable region length between the two datasets. (PDF 55 kb)

Additional file 7 OTU clustering methods perform variably when OTUs ≤ 1 read are culled. When 8 methods were used on a control community of known composition, many reported vastly increased OTU counts compared to known sample diversity (*n*=20, dotted line). Singletons and non-bacterial sequences were removed as part of sequence processing. The dotted line indicates the expected number of OTUs. (PDF 122 kb)

Additional file 8 OTU clustering methods perform variably when all OTUs are included. As visualized in Fig. 4, the number of observed OTUs varies depending on clustering approach. Variability is also observed between sequencing and PCR replicates. OTUs not recognized as Bacteria were removed prior to analysis. (PDF 121 kb)

Additional file 9 Swarm also over-estimates sample diversity. A. When sl1p-generated quality filtered reads were used to pick OTUs with the Swarm algorithm, it also over-estimated within-sample diversity. B. However, many of these spurious OTUs are singletons, indicated by the decrease in the number of OTUs per sample after singletons are removed. (PDF 111 kb)

Additional file 10 The number of observed OTUs converges on the expected community composition as low-abundance OTUs are removed. OTUs with less than n reads were removed (*n*=2 to *n*=10); as n increases, the number of observed OTUs decreases towards the known sample diversity (dotted lined). (PDF 123 kb)

Additional file 11 Taxa present in the taxonomic assignment of HMP-mock1. For the first HMP mock community, the genus-level taxonomic assignments are compared to the known mock community in terms of taxonomic assignment and estimated proportions. Mis-assigned taxa are identified with overlaid patterns. (PDF 141 kb)

Additional file 12 Taxon assignment of HMP-mock2. Taxa were assigned to OTUs resulting from sl1p’s options for OTU clustering, taxon assignment, and choice of reference database. Resulting taxa was compared to the known composition of the community to determine correct taxa assignment. (PDF 153 kb)

Additional file 13 Effects of data processing and PCR/sequencing replicates on *α* diversity metrics. Samples from the HMP-mock community were used to calculate Shannon, Chao1, and Simpson measures of *α* diversity. Together with Fig. 6, these results indicate that choice of OTU clustering algorithm creates large variability in the resulting diversity output. Further, variation is observed across PCR and sequencing replicates (A), which is only partially mitigated by use of rarefaction (B). (PDF 152 kb)
