# Supplementary material for: A comprehensive evaluation of the sl1p pipeline for 16S rRNA gene sequencing analysis
Source: Microbiome. 2017 Aug 14;5:100. doi: 10.1186/s40168-017-0314-2 (PMC5557527; doi:10.1186/s40168-017-0314-2)
Supplement: Supplementary file 1 — Full schematic of the sl1p software. sl1p is a pipeline script which calls a series of sub-routines based on user-defined parameters given as a series of runtime flags. This Fig. accompanies Fig. 1. (PDF 126 kb) [file 40168_2017_314_MOESM1_ESM.pdf]

Usage: sl1p.pl <# of fofns> <list of fofns> <project name> [-r region] [-s seq info file] [-b barcode location] [-u threads] [-f force overwrite] [-q quality threshold] [-m chimera checking] [-p OTU picking algorithm] [-c OTU picking threshold] [-l linkage method] [-t taxonomic assignment method] [-d reference database] [-x timed]

input parameters
-r region of 16S [v3/v34/other] (default: v3)
-s seq info file to use with -r other (default: none)
-b location of barcode on sequence [fwd/rev] (default: fwd)
-u number of threads [0-9] (default: 1)
-f force overwrite output from previous runs [y/n] (default: n)

quality filtering
-q quality threshold [0-40] (default: 30)
-m chimera checking [y/n] (default: y)

OTU clustering
-p OTU picking algorithm [abundantotu/uclust/cdhit/dnaclust/uclust-ref/uclust-ref-strict/mothur/blast/uparse/all
-c OTU picking clustering threshold [0-100] (default: 97)
-l linkage method to use with -c mothur

taxonomic assignment
-t taxonomic assignment method [blast/rdp-training/all] (default: rdp-training)
-d reference database [gg2011/gg2013/silva111/all] (default: gg2011)

output parameters
-x output CPU timing of each data processing step [y/n] (default: n)

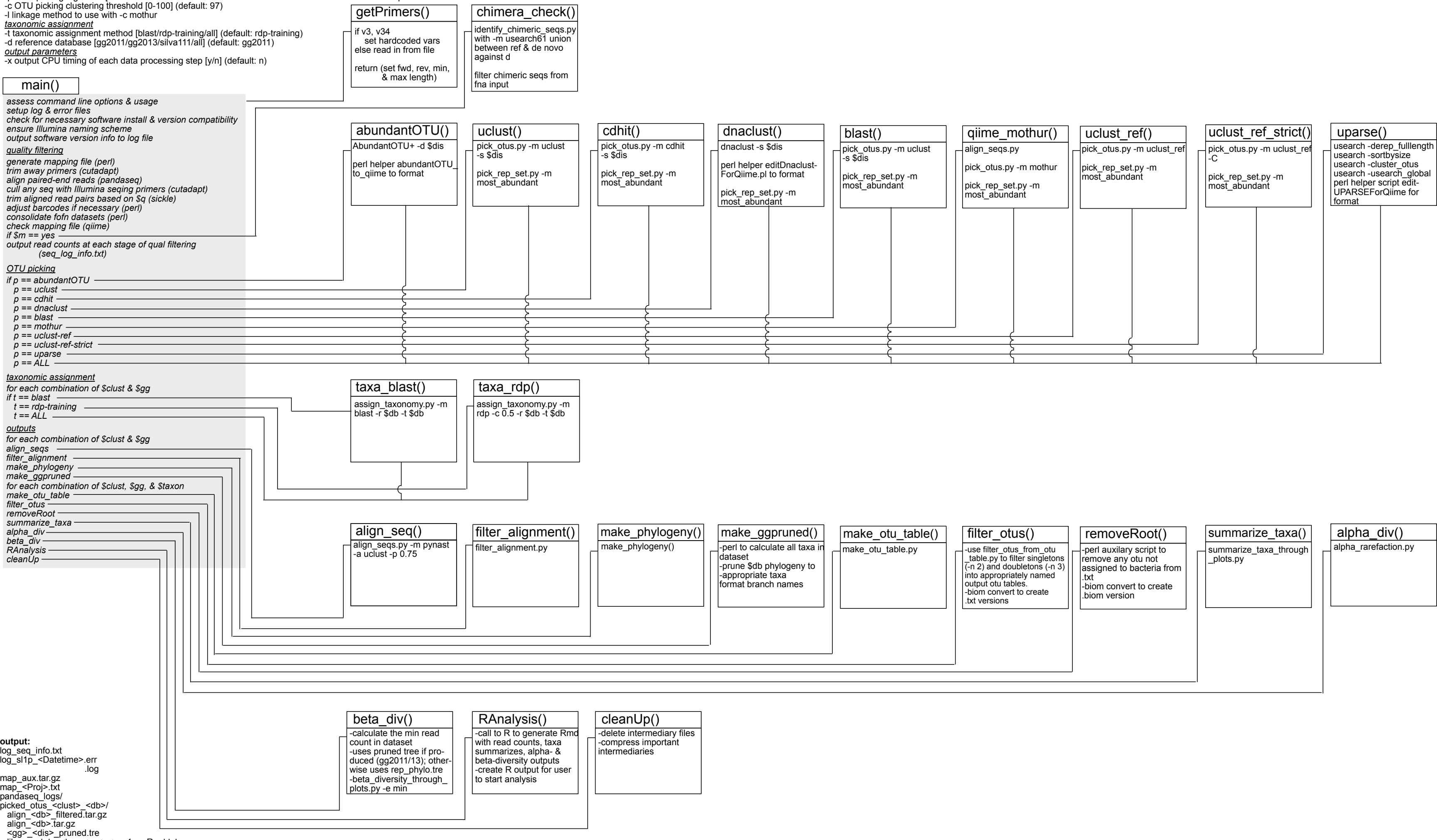

output:
log_seq_info.txt
log_sl1p_<Datetime>.err
.log
map_aux.tar.gz
map_<Proj>.txt
pandaseq_logs/
picked_otus_<clust>_<db>/
align_<db>_filtered.tar.gz
align_<db>.tar.gz
<gg>_<dis>_pruned.tre
library_stats_<taxon>_<gg>_n1_noRoot.txt
library_stats_<taxon>_<gg>.txt
otus_aux.tar.gz
otu_table_<taxon>_<gg>.biom
otu_table_<taxon>_<gg>_n1_noRoot.biom
otu_table_<taxon>_<gg>_n1_noRoot_relAbund.txt
otu_table_<taxon>_<gg>_n1_noRoot.txt
otu_table_<taxon>_<gg>.txt
phylo_aux.tar.gz
<taxon>_<db>_assigned_taxonomy_tr.tar.gz
rep_set_aux.tar.gz
seqs_<db>_otus.txt.tar.gz
sl1p_analysis.html
sl1p_analysis.Rmd
wf_taxa_summary_<taxon>_<db>
wf_taxa_summary_<taxon>_<db>_n1_noRoot
<Proj>.fna.tar.gz
qiime_params.txt
splits_dir.tar.gz
