# Supplementary material for: A comprehensive evaluation of the sl1p pipeline for 16S rRNA gene sequencing analysis
Source: Microbiome. 2017 Aug 14;5:100. doi: 10.1186/s40168-017-0314-2 (PMC5557527; doi:10.1186/s40168-017-0314-2)
Supplement: Supplementary file 2 — R markdown output of the data processing present within this manuscript. This file is in HTML format and displays the output generated by Additional file 3. (HTML 10,148 kb) [file 40168_2017_314_MOESM2_ESM.html]

sl1p\_manuscript\_analyses


# sl1p\_manuscript\_analyses

Setup:

```
packageVersion("ggplot2")
```

```
## [1] '2.1.0'
```

```
packageVersion("cowplot")
```

```
## [1] '0.6.3'
```

```
packageVersion("data.table")
```

```
## [1] '1.9.6'
```

```
packageVersion("phyloseq")
```

```
## [1] '1.16.2'
```

```
packageVersion("plyr")
```

```
## [1] '1.8.4'
```

```
packageVersion("phytools")
```

```
## [1] '0.5.64'
```

```
packageVersion("vegan")
```

```
## [1] '2.4.1'
```

```
packageVersion("MASS")
```

```
## [1] '7.3.45'
```

## HMP-mock data

Setup:

Figure 3a:

Figure 3b:

Sup Fig 5:

Sup Fig 6:

Setup:

Count number of OTUs > 0-10:

Figure 4a, HMP-mock1:

Figure 4a, HMP-mock2:

Sup Fig 7, n=1:

Sup Fig 8, n=0:

Sup Fig 9:

```
## quartz_off_screen 
##                 2
```

Sup Fig 10:

Setup:

Figure 5a:

Sup Fig 11:

Sup Fig 12:

Figure 6, expected values:

```
## Processing map file...
## Processing otu/tax file...
## Reading file into memory prior to parsing...
## Detecting first header line...
## Header is on line 2  
## Converting input file to a table...
## Defining OTU table... 
## Parsing taxonomy table...
```

Figure 6a:

```
## quartz_off_screen 
##                 2
```

Figure 6b:

Figure 6c:

Figure 6d (perl code here for completeness):

Sup Fig 13a:

Sup Fig 13b:

## URTCul-Single data

Setup:

Figure 4b:

Figure 5b: Initialize function from http://stackoverflow.com/questions/13673894/suppress-nas-in-paste

## URTCul-combined

Setup:

Figure 4c, URT-combined

Figure 5c, URT-combined

## URTCul-unique

Setup:

Figure 4c, URT-unique

Figure 5c, URT-unique
