# Supplementary material for: A comprehensive evaluation of the sl1p pipeline for 16S rRNA gene sequencing analysis
Source: Microbiome. 2017 Aug 14;5:100. doi: 10.1186/s40168-017-0314-2 (PMC5557527; doi:10.1186/s40168-017-0314-2)
Supplement: Supplementary file 4 — The R markdown output generated by sl1p for the HMP-mock dataset. (HTML 1546 kb) [file 40168_2017_314_MOESM4_ESM.html]

# Sl1p Preliminary Analyses

## Author: Fiona J. Whelan

### whelanfj@mcmaster.ca

### (included R markdown and html output for the user’s convenience)

#### Load phyloseq object. This script uses phyloseq (https://joey711.github.io/phyloseq/) to input the OTU table, map file, and phylogeny from sl1p into R.

```
qd
```

```
## phyloseq-class experiment-level object
## otu_table()   OTU Table:         [ 276 taxa and 13 samples ]
## sample_data() Sample Data:       [ 13 samples by 4 sample variables ]
## tax_table()   Taxonomy Table:    [ 276 taxa by 6 taxonomic ranks ]
## phy_tree()    Phylogenetic Tree: [ 276 tips and 211 internal nodes ]
```

#### Preliminary Analyses

#### 1. Observe data for non-bacterial contamination

```
L2 <- read.table(L2File, sep="\t", comment.char = '&', header=TRUE, row.names=1, skip=1)
L2 <- t(L2)
#Order by abundance
L2 <- L2[,order(colSums(L2), decreasing=TRUE)]
#Change any individual values <1.0% to 0
L2[L2<0.01] <- 0
#Remove any taxa that now sum to 0
L2 <- L2[,colSums(L2)>0]
#Add an Others column
L2 <- as.data.frame(L2)
L2[,"Taxa < 1%"] <- 1-rowSums(L2)
#Assign colours using function
colours <- myColours(colnames(L2))
#Plot
df <- NULL
for (i in 1:dim(L2)[2]){
        tmp <- data.frame(row.names=NULL, Sample=rownames(L2), Taxa=rep(colnames(L2)[i], dim(L2)[1]), Value=L2[,i])
        if(i==1){df <- tmp} else {df <- rbind(df, tmp)}
}
p <- ggplot(df, aes(Sample,Value,fill=Taxa))
p <- p + geom_bar(stat="identity")
p <- p + theme_bw()
p <- p + ylab("Proportions")
p <- p + scale_fill_manual(values=colours)
p <- p + scale_y_continuous(expand = c(0,0))
p <- p + theme(axis.text.x=element_text(angle=90,hjust=1,vjust=0.5))
p <- p + guides(fill=guide_legend(ncol=1))
p
```

#### 2. Observe filtered sequencing depth

```
## Num samples: 13
## Num observations: 540
## Total count: 687121
## Table density (fraction of non-zero values): 0.229
## 
## Counts/sample summary:
##  Min: 39.0
##  Max: 113084.0
##  Median: 35275.000
##  Mean: 52855.462
##  Std. dev.: 36960.186
##  Sample Metadata Categories: None provided
##  Observation Metadata Categories: taxonomy
## 
## Counts/sample detail:
## FW.HMP1.r1.v3.neg: 39.0
## FW.HMP2.r1.v3.3: 5917.0
## FW.HMP2.r1.v3.1: 25511.0
## FW.HMP1.r1.v3.2: 27951.0
## FW.HMP1.r1.v3.1: 30250.0
## FW.HMP2.r1.v3.2: 32492.0
## FW.HMP1.r1.v3.3: 35275.0
## FW.HMP2.r2.v3.3: 48662.0
## FW.HMP1.r2.v3.1: 80731.0
## FW.HMP2.r2.v3.1: 85137.0
## FW.HMP1.r2.v3.3: 95731.0
## FW.HMP1.r2.v3.2: 106341.0
## FW.HMP2.r2.v3.2: 113084.0
```

#### 3. Output taxonomic summary of the data

```
L6 <- read.table(L6File, sep="\t", comment.char = '&', header=TRUE, row.names=1, skip=1)
L6 <- t(L6)
#Order by abundance
L6 <- L6[,order(colSums(L6), decreasing=TRUE)]
#Change any individual values <1.0% to 0
L6[L6<0.01] <- 0
#Remove any taxa that now sum to 0
L6 <- L6[,colSums(L6)>0]
#Add an Others column
L6 <- as.data.frame(L6)
L6[,"Taxa < 1%"] <- 1-rowSums(L6)
#Assign colours using function
colours <- myColours2(ncol(L6))
#Plot
df <- NULL
for (i in 1:dim(L6)[2]){
    tmp <- data.frame(row.names=NULL, Sample=rownames(L6), Taxa=rep(colnames(L6)[i], dim(L6)[1]), Value=L6[,i])
    if(i==1){df <- tmp} else {df <- rbind(df, tmp)}
}
p <- ggplot(df, aes(Sample,Value,fill=Taxa))
p <- p + scale_fill_manual(values=colours)
p <- p + geom_bar(stat="identity")
p <- p + theme_bw()
p <- p + ylab("Proportions")
p <- p + scale_y_continuous(expand = c(0,0))
p <- p + theme(axis.text.x=element_text(angle=90,hjust=1,vjust=0.5))
p <- p + guides(fill=guide_legend(ncol=1))
p
```

#### Do proportional normalization

Code a modified form of that supplied in the supplemental data of Waste Not, Want No

```
normf = function(x) {
  x/sum(x)
}
qd.norm = transform_sample_counts(qd, normf)
```

#### Do rarefication normalization

```
rare.depth = min(sample_sums(qd))
qd.rare = rarefy_even_depth(qd, sample.size = rare.depth, rngseed=1414)
```

#### 4. Output alpha diversity metrics

```
p1 <- plot_richness(qd, x="X.SampleID", measures=c("Shannon", "Simpson", "Chao1"))
p1 <- p1 + ggtitle("Unadjusted Alpha Diversity scores")
p1
```

```
## Warning: Removed 26 rows containing missing values (geom_errorbar).
```

```
p2 <- plot_richness(qd.rare, x="X.SampleID", measures=c("Shannon", "Simpson", "Chao1"))
p2 <- p2 + ggtitle(paste("Rarefied (n=",rare.depth,") Alpha Diversity scores", sep=""))
p2
```

```
## Warning: Removed 26 rows containing missing values (geom_errorbar).
```

```
#p <- plot_grid(p1, p2, labels=c("A", "B"), ncol = 1, nrow = 2)
#p
#ggsave(file="alpha.eps", p, scale=4)
```

#### 5. Output beta diversity metrics

```
#Unadjusted
dist_bray <- phyloseq::distance(qd, method = "bray")
iMDS_bray <- ordinate(qd, "PCoA", distance = dist_bray)
dist_unwe <- phyloseq::distance(qd, method = "unifrac")
iMDS_unwe <- ordinate(qd, "PCoA", distance = dist_unwe)
dist_weun <- phyloseq::distance(qd, method = "wunifrac")
iMDS_weun <- ordinate(qd, "PCoA", distance = dist_weun)
p1 <- plot_ordination(qd, iMDS_bray)
p1 <- p1 + ggtitle(paste("Unadjusted Bray Curtis", sep=""))
p1 <- p1 + geom_point(aes(color=X.SampleID), pch=16, size=3)
p1 <- p1 + theme_bw()
p2 <- plot_ordination(qd, iMDS_unwe)
p2 <- p2 + ggtitle(paste("Unadjusted Unweighted Unifrac", sep=""))
p2 <- p2 + geom_point(aes(color=X.SampleID), pch=16, size=3)
p2 <- p2 + theme_bw()
p3 <- plot_ordination(qd, iMDS_weun)
p3 <- p3 + ggtitle(paste("Unadjusted Weighted Unifrac", sep=""))
p3 <- p3 + geom_point(aes(color=X.SampleID), pch=16, size=3)
p3 <- p3 + theme_bw()
p <- plot_grid(p1, p2, p3, labels=c("A", "B", "C"), ncol=3, nrow=1)
p
```

```
#Rarefied
Rdist_bray <- phyloseq::distance(qd.rare, method = "bray")
RiMDS_bray <- ordinate(qd.rare, "PCoA", distance = Rdist_bray)
Rdist_unwe <- phyloseq::distance(qd, method = "unifrac")
RiMDS_unwe <- ordinate(qd.rare, "PCoA", distance = Rdist_unwe)
Rdist_weun <- phyloseq::distance(qd.rare, method = "wunifrac")
RiMDS_weun <- ordinate(qd.rare, "PCoA", distance = Rdist_weun)
p4 <- plot_ordination(qd.rare, RiMDS_bray)
p4 <- p4 + ggtitle(paste("Rarefied (n=",rare.depth,") Bray Curtis"))
p4 <- p4 + geom_point(aes(color=X.SampleID), pch=16, size=3)
p4 <- p4 + theme_bw()
p5 <- plot_ordination(qd.rare, RiMDS_unwe)
p5 <- p5 + ggtitle(paste("Rarefied (n=",rare.depth,") Unweighted Unifrac"))
p5 <- p5 + geom_point(aes(color=X.SampleID), pch=16, size=3)
p5 <- p5 + theme_bw()
p6 <- plot_ordination(qd.rare, RiMDS_weun)
p6 <- p6 + ggtitle(paste("Rarefied (n=",rare.depth,") Weighted Unifrac"))
p6 <- p6 + geom_point(aes(color=X.SampleID), pch=16, size=3)
p6 <- p6 + theme_bw()
p <- plot_grid(p4, p5, p6, labels=c("A", "B", "C"), ncol = 3, nrow = 1)
p
```

```
#ggsave(file="beta.eps", p, scale=4)
```
