# Supplementary figures and images for: A comprehensive evaluation of the sl1p pipeline for 16S rRNA gene sequencing analysis
Source: Microbiome. 2017 Aug 14;5:100. doi: 10.1186/s40168-017-0314-2 (PMC5557527; doi:10.1186/s40168-017-0314-2)

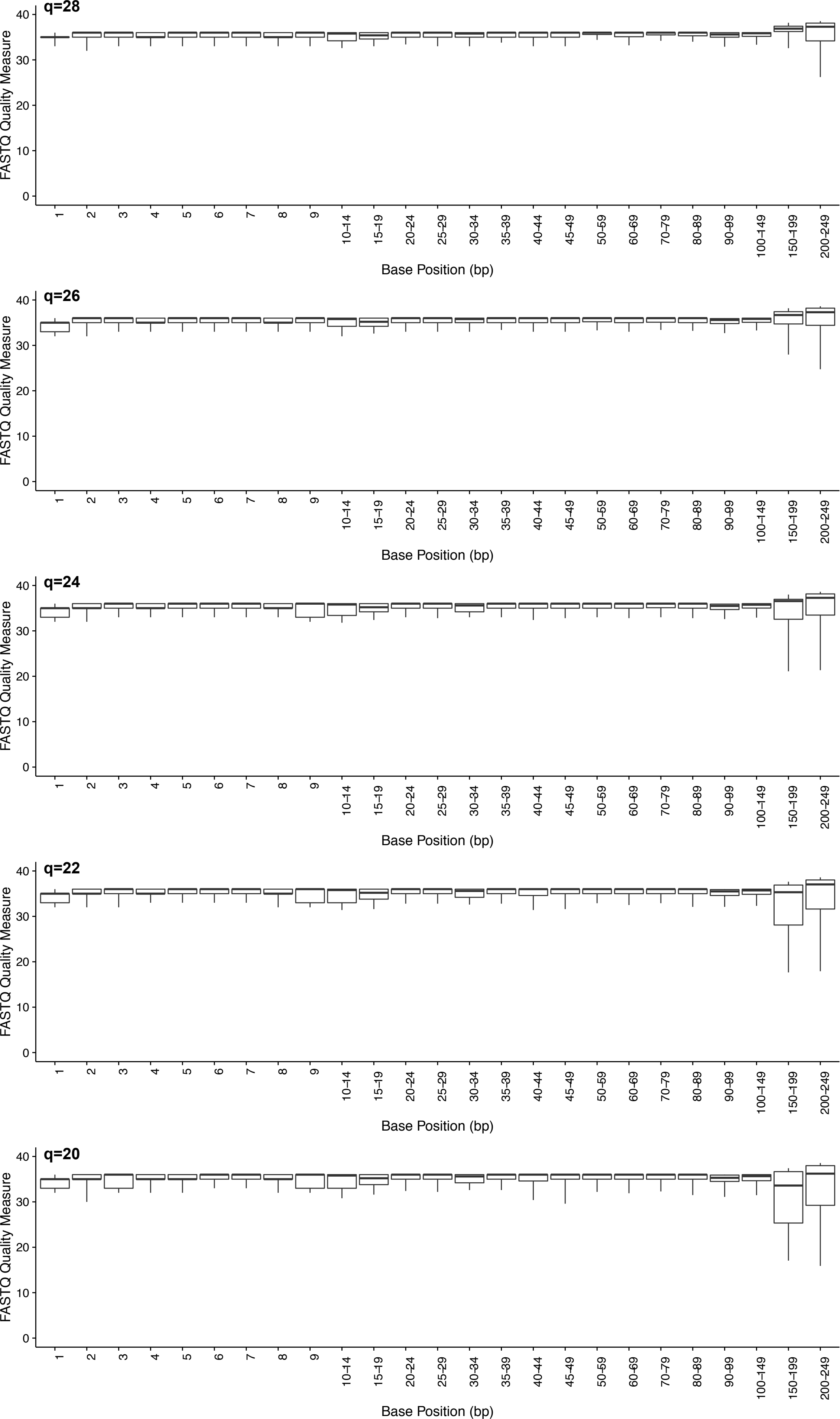

Supplement: Supplementary file 5 — Comparisons of various thresholds for quality trimming. Sickle takes as input a quality threshold with which it determines its quality trimming parameters. Here, we compare the results with a threshold of 30 (Fig. 3) with sequentially lower quality threshold inputs into sickle. (PDF 40 kb) [file 40168_2017_314_MOESM5_ESM.pdf]

# IDSeq

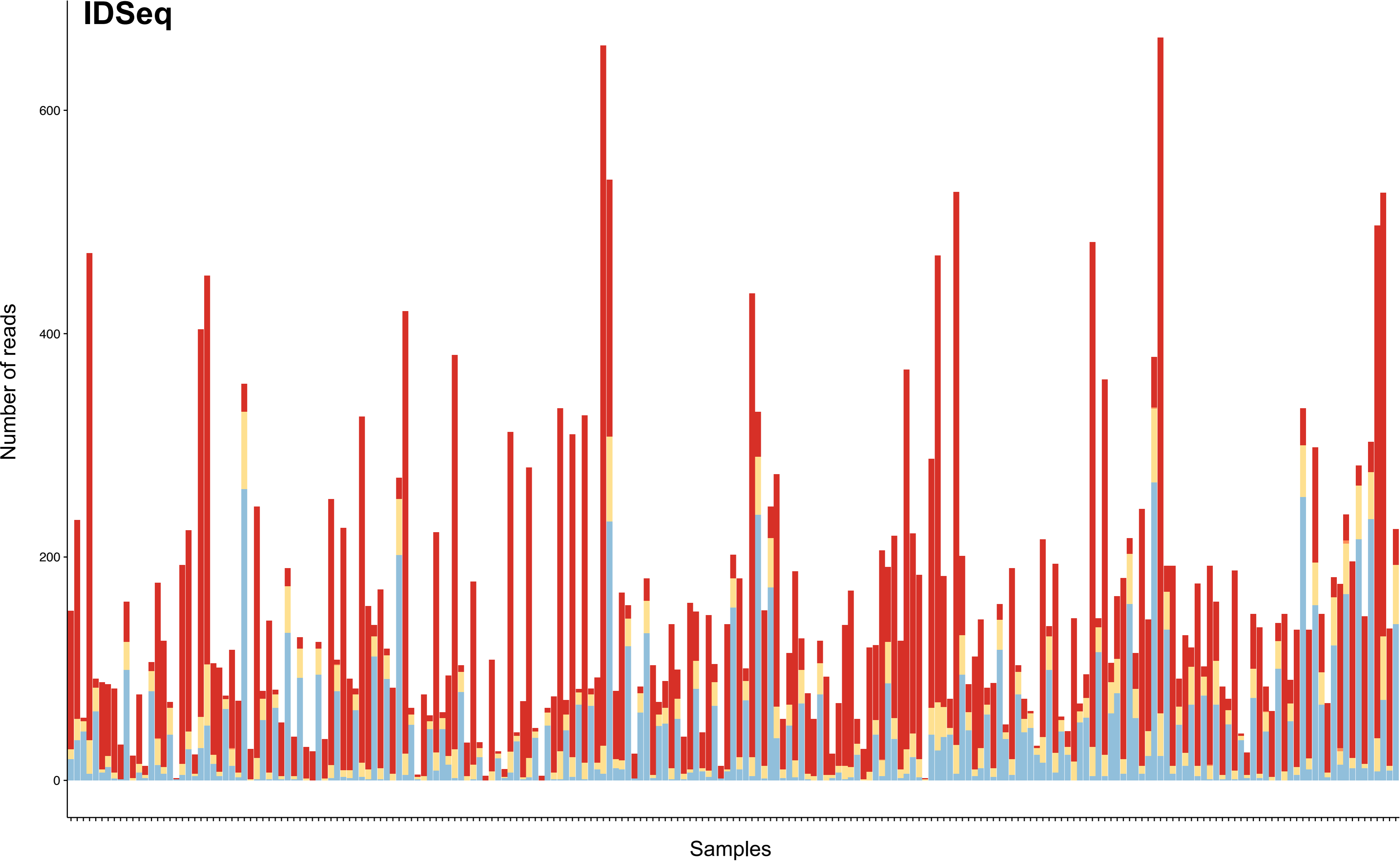

## Legend

Paired-end assembly Residual primers Quality filtering (Q=30) Passed reads

Supplement: Supplementary file 6 — Outline of reads lost in the URTCul dataset during sl1p’s quality control pipeline. More input reads were culled during the PANDAseq alignment step in this dataset compared to HMP-mock (Fig. 3), possibly due to a difference in target variable region length between the two datasets. (PDF 55 kb) [file 40168_2017_314_MOESM6_ESM.pdf]

**A. n=0**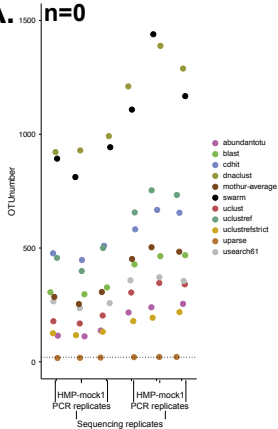**n=0**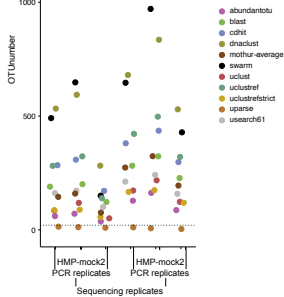**B.**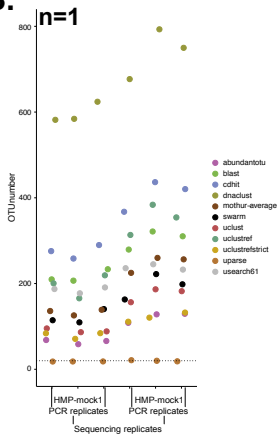**n=1**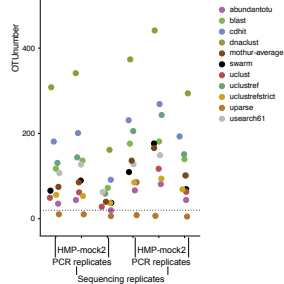

Supplement: Supplementary file 9 — Swarm also over-estimates sample diversity. A. When sl1p-generated quality filtered reads were used to pick OTUs with the Swarm algorithm, it also over-estimated within-sample diversity. B. However, many of these spurious OTUs are singletons, indicated by the decrease in the number of OTUs per sample after singletons are removed. (PDF 111 kb) [file 40168_2017_314_MOESM9_ESM.pdf]

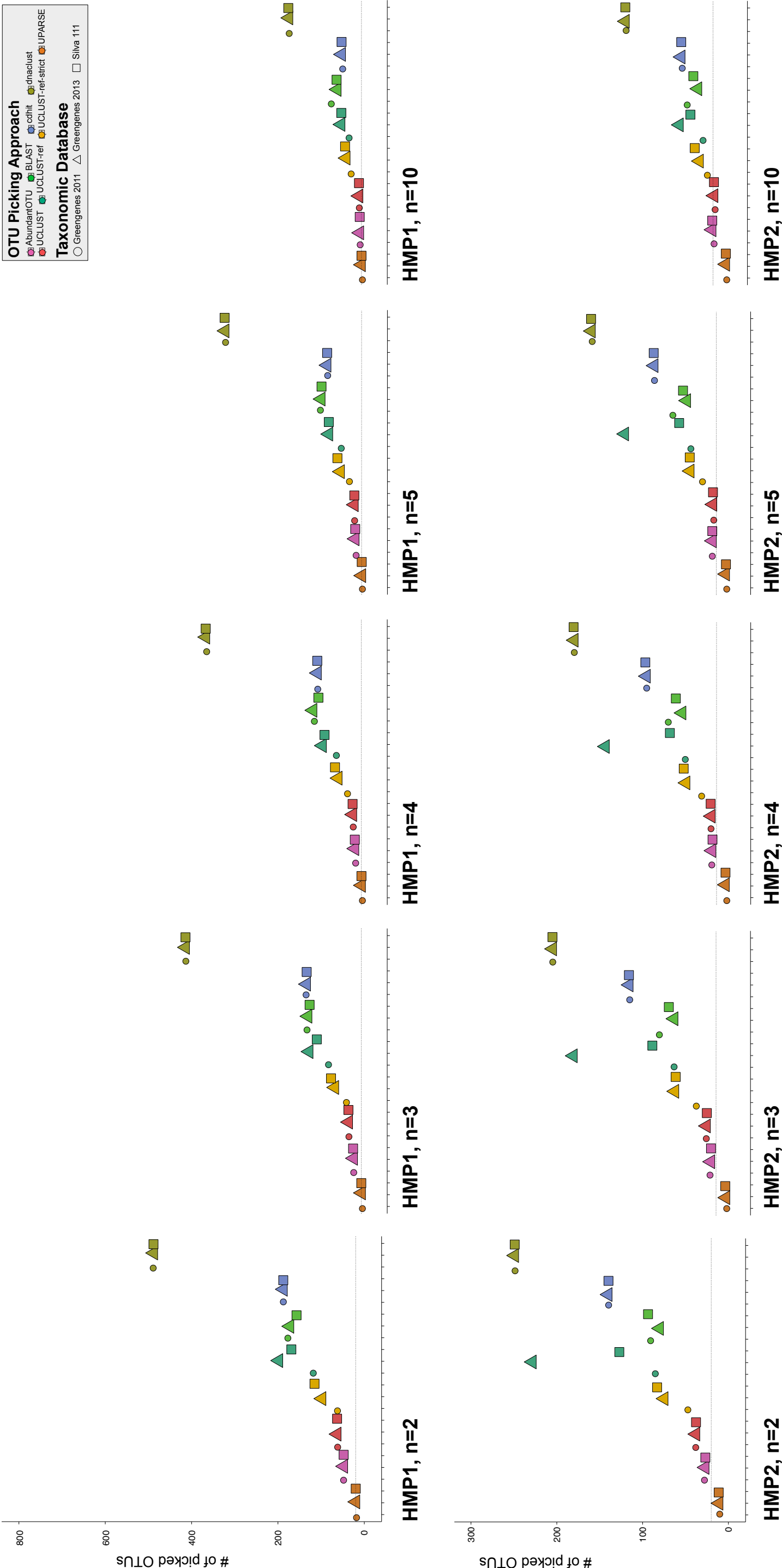

Supplement: Supplementary file 10 — The number of observed OTUs converges on the expected community composition as low-abundance OTUs are removed. OTUs with less than n reads were removed (n=2 to n=10); as n increases, the number of observed OTUs decreases towards the known sample diversity (dotted lined). (PDF 123 kb) [file 40168_2017_314_MOESM10_ESM.pdf]

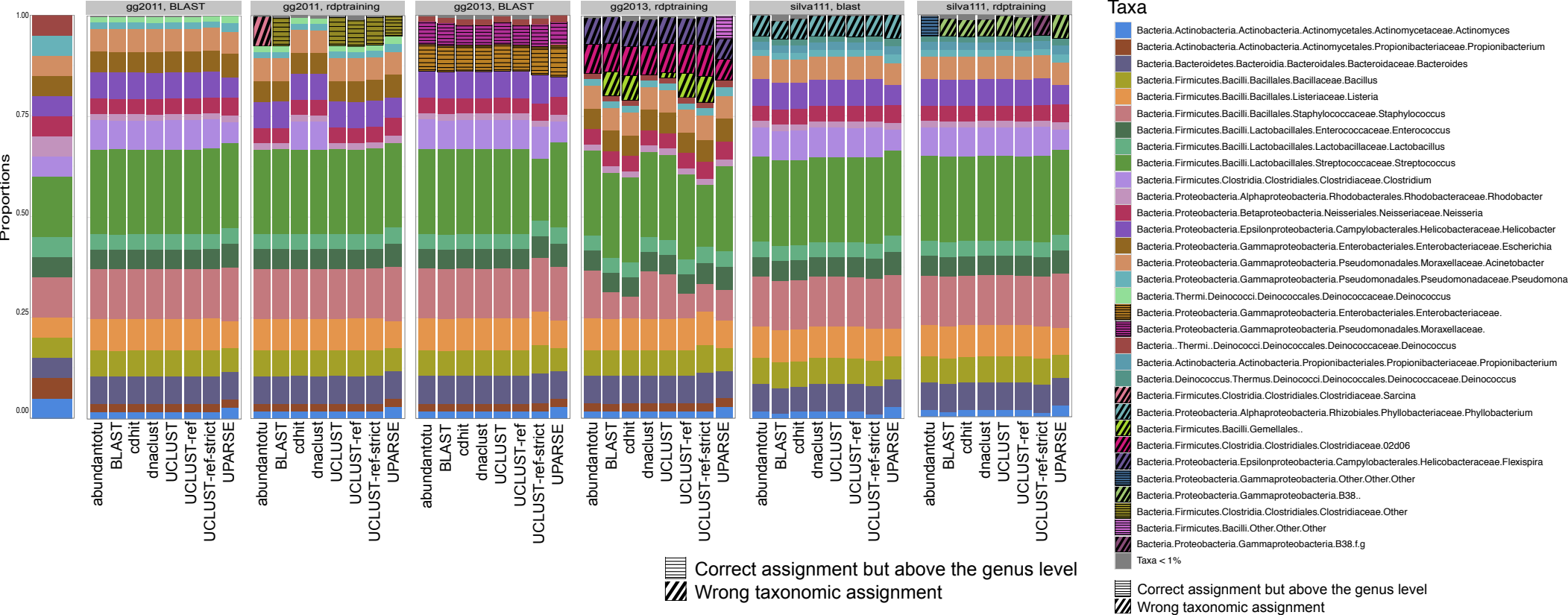

Supplement: Supplementary file 11 — Taxa present in the taxonomic assignment of HMP-mock1. For the first HMP mock community, the genus-level taxonomic assignments are compared to the known mock community in terms of taxonomic assignment and estimated proportions. Mis-assigned taxa are identified with overlaid patterns. (PDF 141 kb) [file 40168_2017_314_MOESM11_ESM.pdf]

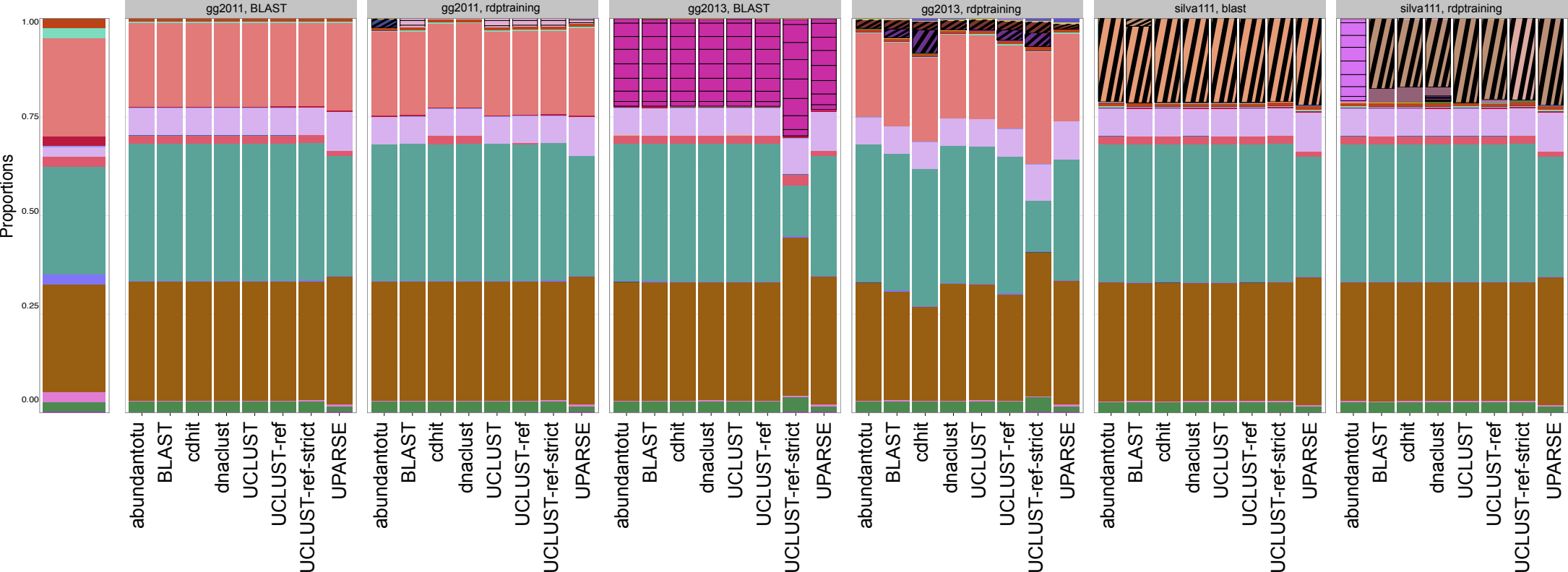

### Taxa

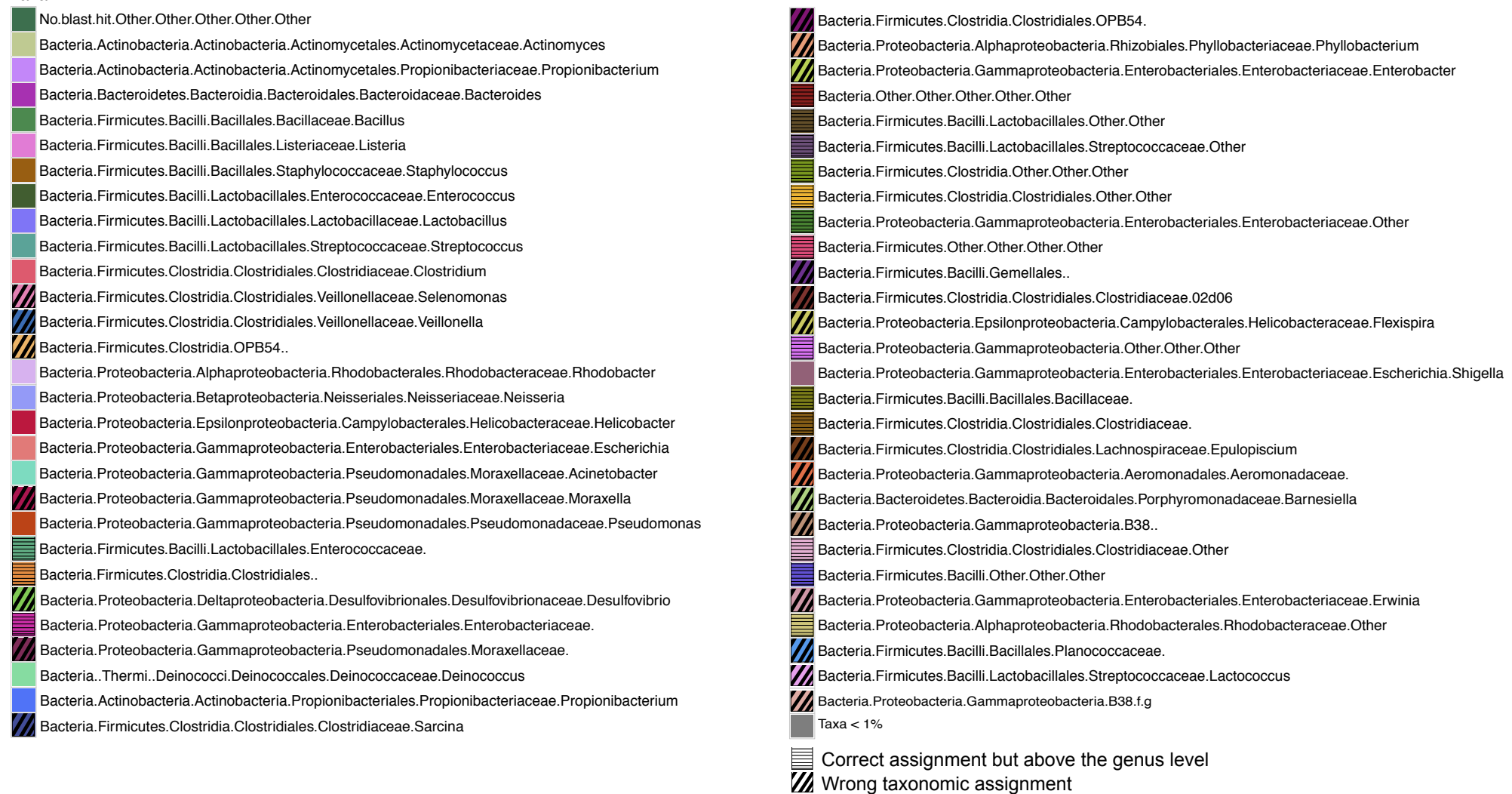

Supplement: Supplementary file 12 — Taxon assignment of HMP-mock2. Taxa were assigned to OTUs resulting from sl1p’s options for OTU clustering, taxon assignment, and choice of reference database. Resulting taxa was compared to the known composition of the community to determine correct taxa assignment. (PDF 153 kb) [file 40168_2017_314_MOESM12_ESM.pdf]
