# Supplementary material for: A comprehensive evaluation of the sl1p pipeline for 16S rRNA gene sequencing analysis
Source: Microbiome. 2017 Aug 14;5:100. doi: 10.1186/s40168-017-0314-2 (PMC5557527; doi:10.1186/s40168-017-0314-2)
Supplement: Supplementary file 7 — OTU clustering methods perform variably when OTUs ≤ 1 read are culled. When 8 methods were used on a control community of known composition, many reported vastly increased OTU counts compared to known sample diversity (n=20, dotted line). Singletons and non-bacterial sequences were removed as part of sequence processing. The dotted line indicates the expected number of OTUs. (PDF 122 kb) [file 40168_2017_314_MOESM7_ESM.pdf]

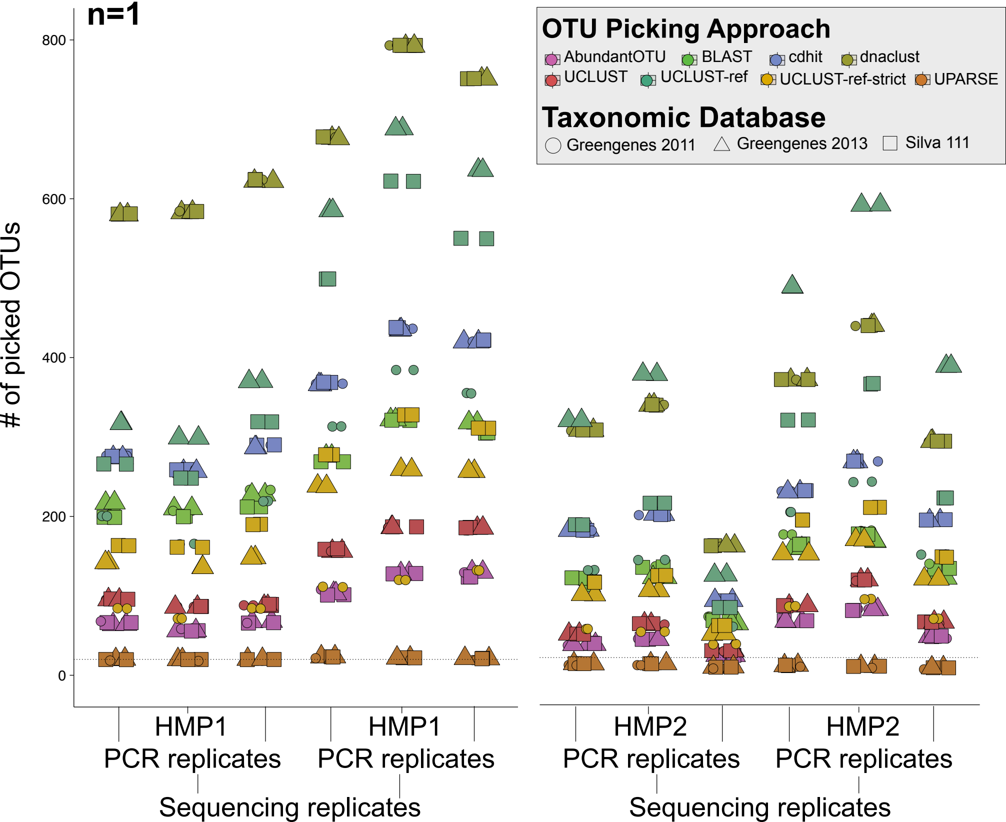

n=1
OTU Picking Approach
AbundantOTU
BLAST
cdhit
dnaclust
UCLUST
UCLUST-ref
UCLUST-ref-strict
UPARSE
Taxonomic Database
Greengenes 2011
Greengenes 2013
Silva 111
# of picked OTUs
800
600
400
200
0
HMP1
PCR replicates
HMP1
PCR replicates
Sequencing replicates
HMP2
PCR replicates
HMP2
PCR replicates
Sequencing replicates
