# Supplementary material for: A comprehensive evaluation of the sl1p pipeline for 16S rRNA gene sequencing analysis
Source: Microbiome. 2017 Aug 14;5:100. doi: 10.1186/s40168-017-0314-2 (PMC5557527; doi:10.1186/s40168-017-0314-2)
Supplement: Supplementary file 8 — OTU clustering methods perform variably when all OTUs are included. As visualized in Fig. 4, the number of observed OTUs varies depending on clustering approach. Variability is also observed between sequencing and PCR replicates. OTUs not recognized as Bacteria were removed prior to analysis. (PDF 121 kb) [file 40168_2017_314_MOESM8_ESM.pdf]

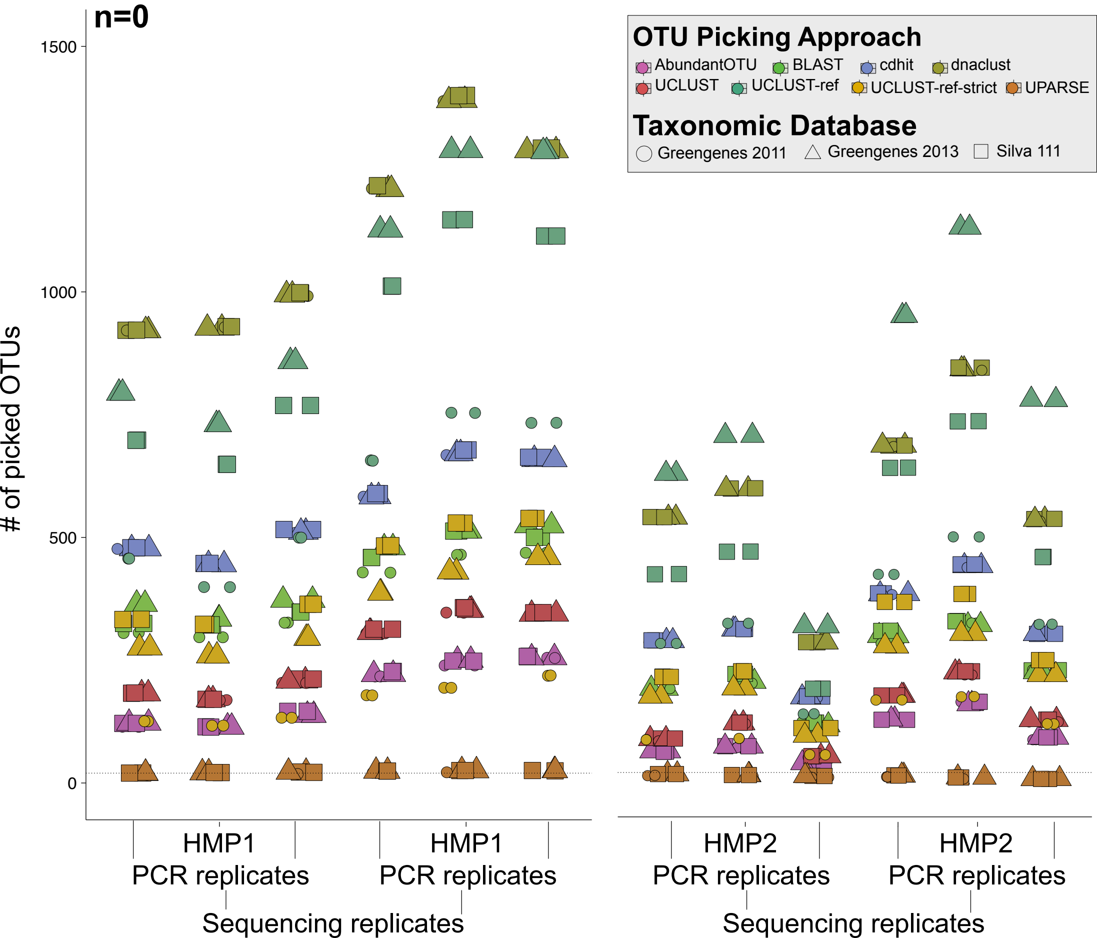
n=0
OTU Picking Approach
AbundantOTU
BLAST
cdhit
dnaclust
UCLUST
UCLUST-ref
UCLUST-ref-strict
UPARSE
Taxonomic Database
Greengenes 2011
Greengenes 2013
Silva 111
# of picked OTUs
1500
1000
500
0
HMP1
PCR replicates
HMP1
PCR replicates
HMP2
PCR replicates
HMP2
PCR replicates
Sequencing replicates
Sequencing replicates
