# Supplementary material for: A comprehensive evaluation of the sl1p pipeline for 16S rRNA gene sequencing analysis
Source: Microbiome. 2017 Aug 14;5:100. doi: 10.1186/s40168-017-0314-2 (PMC5557527; doi:10.1186/s40168-017-0314-2)
Supplement: Supplementary file 13 — Effects of data processing and PCR/sequencing replicates on α diversity metrics. Samples from the HMP-mock community were used to calculate Shannon, Chao1, and Simpson measures of α diversity. Together with Fig. 6, these results indicate that choice of OTU clustering algorithm creates large variability in the resulting diversity output. Further, variation is observed across PCR and sequencing replicates (A), which is only partially mitigated by use of rarefaction (B). (PDF 152 kb) [file 40168_2017_314_MOESM13_ESM.pdf]

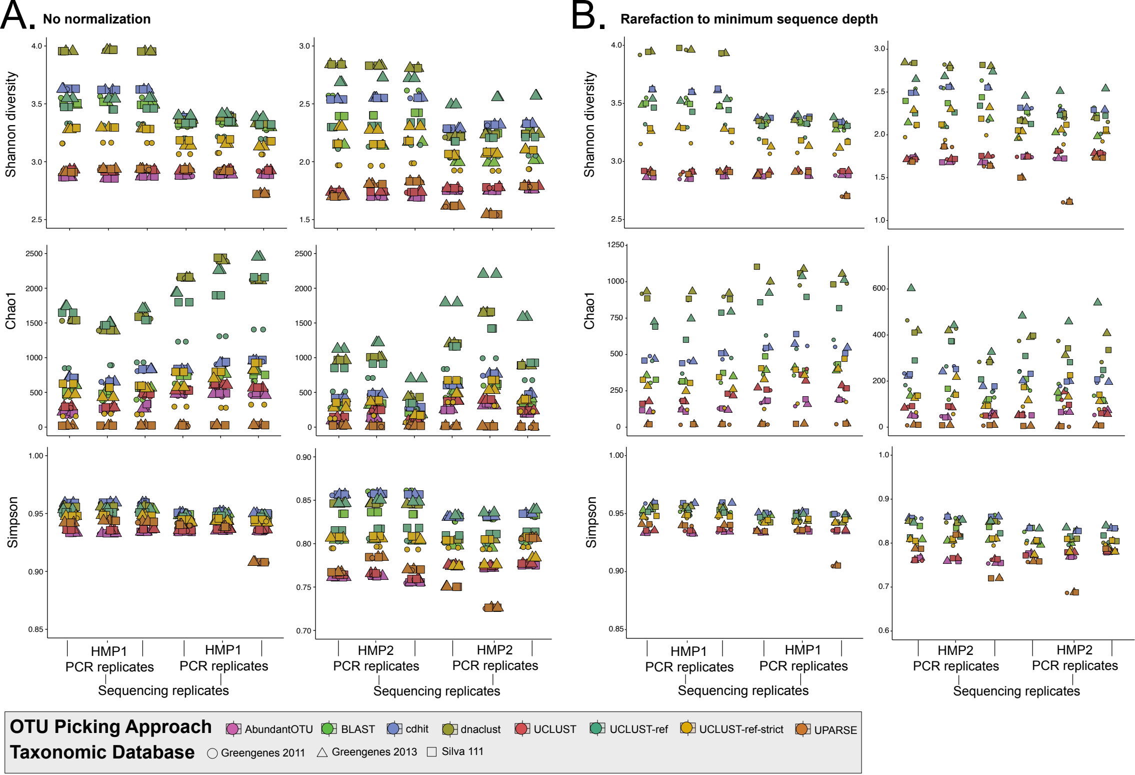
A.
No normalization
B.
Rarefaction to minimum sequence depth
Shannon diversity
Chao1
Simpson
HMP1
PCR replicates
HMP2
Sequencing replicates
OTU Picking Approach
AbundantOTU
BLAST
cdhit
dnaclust
UCLUST
UCLUST-ref
UCLUST-ref-strict
UPARSE
Taxonomic Database
Greengenes 2011
Greengenes 2013
Silva 111
